# Supplementary material for: Enabling fast-charging selenium-based aqueous batteries via conversion reaction with copper ions
Source: Nat Commun. 2022 Apr 6;13:1863. doi: 10.1038/s41467-022-29537-5 (PMC8987094; doi:10.1038/s41467-022-29537-5)
Supplement: Supplementary file 1 — Supplementary Information [file 41467_2022_29537_MOESM1_ESM.pdf]

Supplementary Information for

Enabling fast-charging selenium-based aqueous batteries via conversion reaction with copper ions

Chunlong Dai,[1] Linyu Hu,[1] Hao Chen,[2] Xuting Jin,[1] Yuyang Han,[1] Ying Wang,[1] Xiangyang Li,[1] Xinqun Zhang,[1] Li Song,[1] Maowen Xu,[2] Huhu Cheng,[3] Yang Zhao,[1] Zhipan Zhang,[1] Feng Liu,[4*] Liangti Qu [1,3 *]*

[1] Key Laboratory of Cluster Science, Ministry of Education, Beijing Key Laboratory of Photoelectronic/Electrophotonic Conversion Materials, School of Chemistry and Chemical Engineering, Beijing Institute of Technology, Beijing 100081, P. R. China.
[2] Key Laboratory of Luminescent and Real Time Analytical Chemistry (Southwest University), Ministry of Education, School of Materials and Energy, Southwest University, Chongqing 400715, P. R. China.
[3] Key Laboratory of Organic Optoelectronics & Molecular Engineering of Ministry of Education, Department of Chemistry, Tsinghua University, 100084 Beijing, P. R. China.
[4] State Key Laboratory of Nonlinear Mechanics Institute of Mechanics, Chinese Academy of Sciences, Beijing 100190, P. R. China.

Corresponding authors
Zhipan Zhang: zhipan@bit.edu.cn
Feng Liu: liufeng@imech.ac.cn
Liangti Qu: lqu@mail.tsinghua.edu.cn

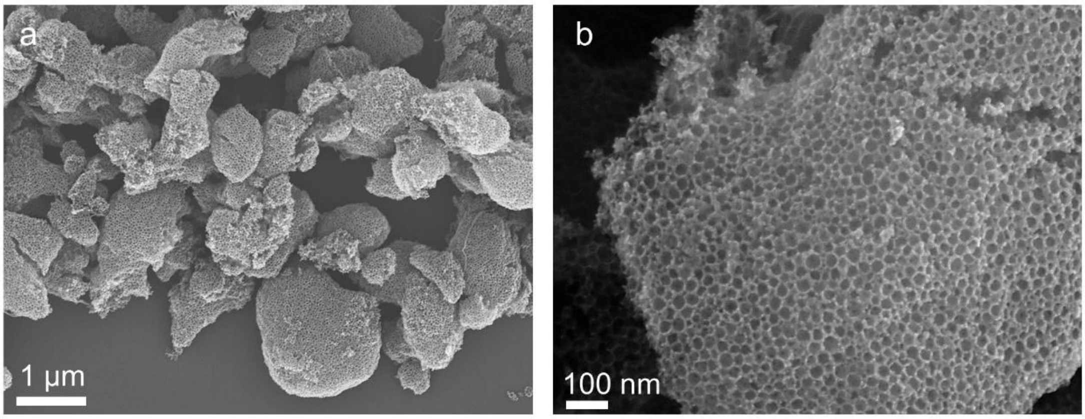

Supplementary Figure 1. SEM images of the porous carbon.

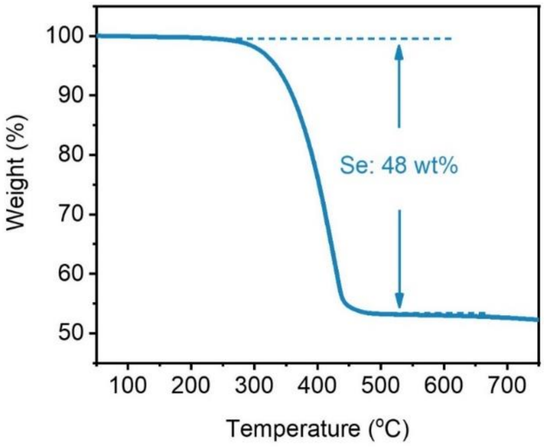

Supplementary Figure 2. The TGA curves of the Se@C-48 composite.

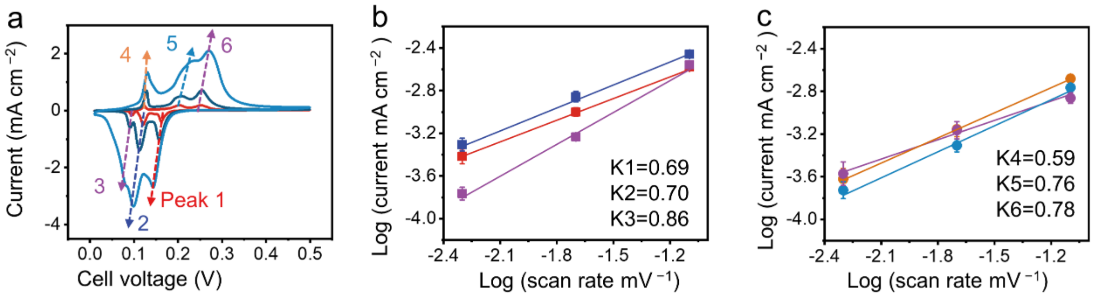

Supplementary Figure 3. a The cyclic voltammetry (CV) curves of Cu|0.5 M $CuSO_4$|Se@C-48 cells at different scan rates of 0.005, 0.02, and 0.08 mV s^{-1}. **b-c** The relationship between current peak currents and scan rates. Error bars represent the standard deviation of five different experiments. The k value of all peaks are larger than 0.5, suggesting all oxidation and reduction reactions are diffusion-controlled.

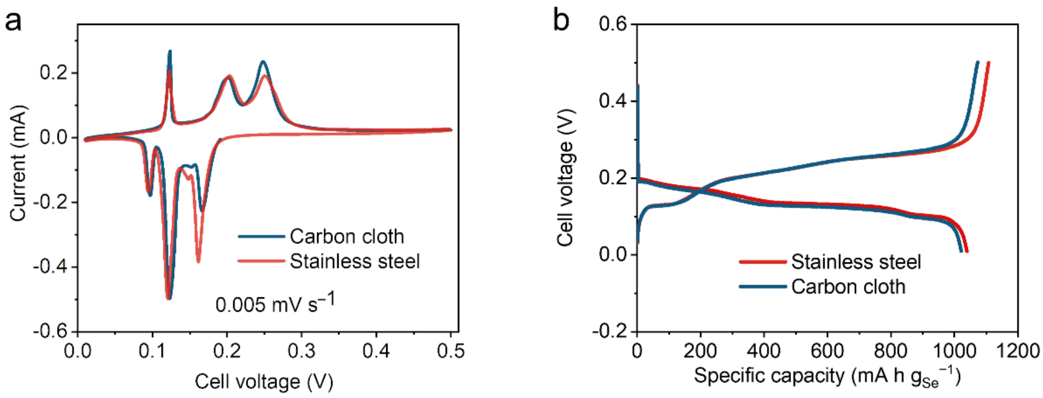

Supplementary Figure 4. **a** Cyclic voltammetry and **b** galvanostatic cycling measurements of the Cu|0.5 M $CuSO_4$|Se@C-48 cells using stainless steel or carbon cloth as cathode current collectors.

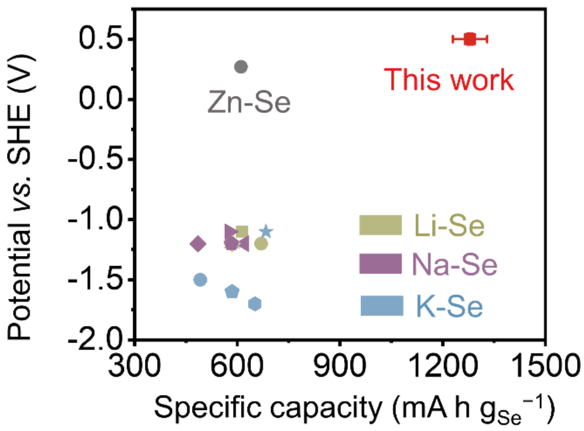

Supplementary Figure 5. The redox potential and specific capacity comparison with other Se-based cathodes; Error bars represent the standard deviation of five different experiments. The specific values are shown in Supplementary Table 1.

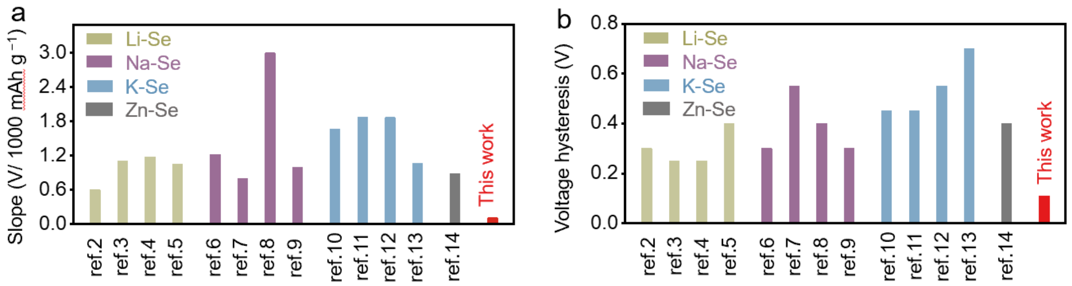

Supplementary Figure 6. The comparisons of **a** discharging slope and **b** charge/discharge voltage hysteresis with other Se-based cells. The specific values are shown in Supplementary Table 1.

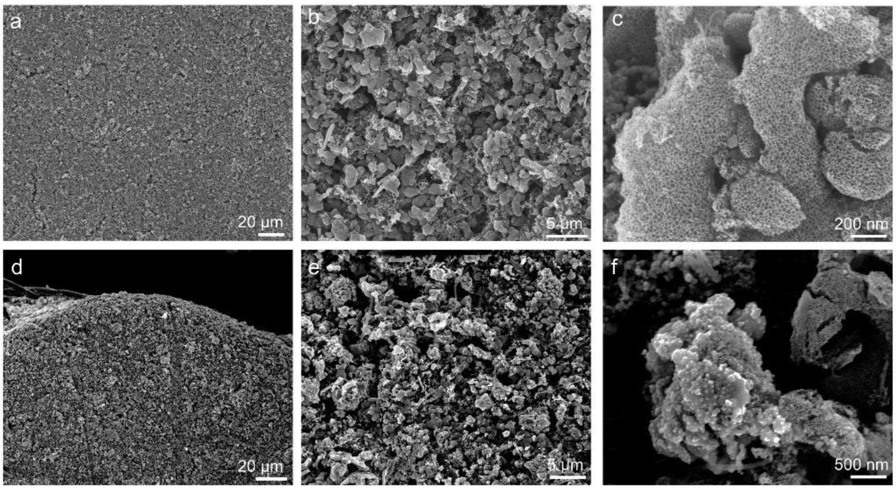

Supplementary Figure 7. SEM images of Se@C-48 electrode **a-c** before and **d-e** after cycled at 5 A g^{-1} for 300 cycles (the voltage range is 0.01 to 0.5 V). The Cu|0.5 M $CuSO_4$|Se@C-48 cell was disconnected at the discharging state.

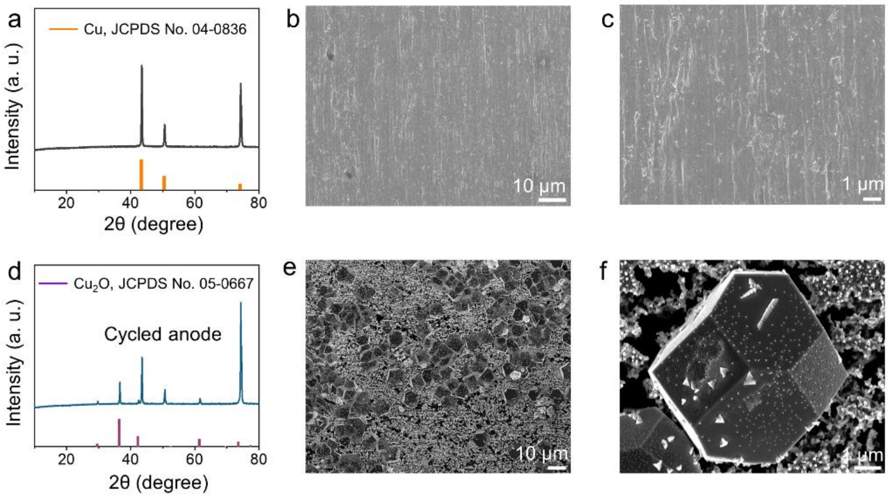

Supplementary Figure 8. a The XRD pattern and **b-c** SEM images of Cu anode before cycling. **d** The XRD pattern and **e-f** SEM images of Cu anode after cycled 300 cycles at 5 A g^{-1}.

The fresh Cu anode is well matched with Cu (JCPDS No. 04-0836) and shows a rough plane structure. After cycled for 300 cycles at 5 A g^{-1}, it changed into Cu/Cu_2O composite. The formation of Cu_2O is mainly attributed to the dissolved oxygen in electrolyte. The SEM images show that some irregular particles with tens of micrometers in size were formed, which should be attributed to the Cu/Cu_2O composite.

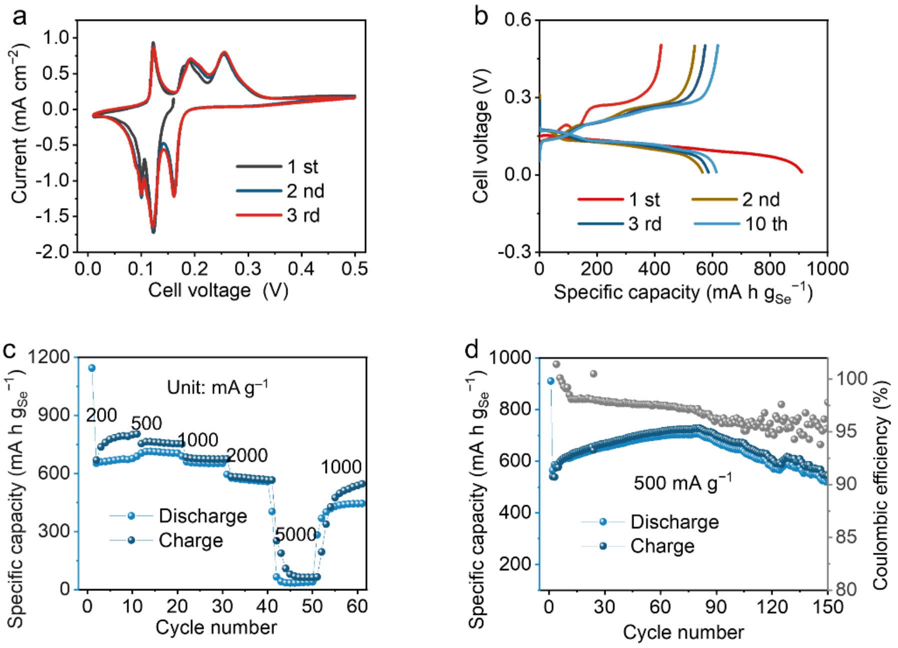

Supplementary Figure 9. The electrochemical performance of Cu|0.5 M $CuSO_4$|Se cell with pure Se as the cathode. The **a** CV curves at 0.02 mV s^{-1}, **b** GCD curves at 500 mA g^{-1}, **c** rate performance, and **d** cycling performance of pure Se as the cathode. The first discharge specific capacity of pure Se cathode is 910 mAh g_{Se}^{-1}.

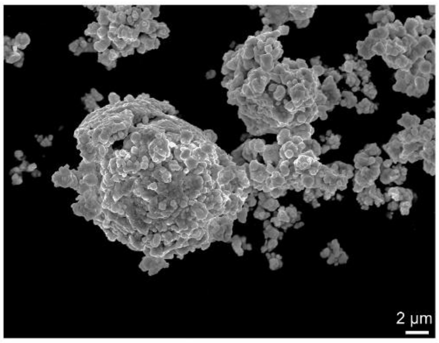

Supplementary Figure 10. SEM image of pure Se.

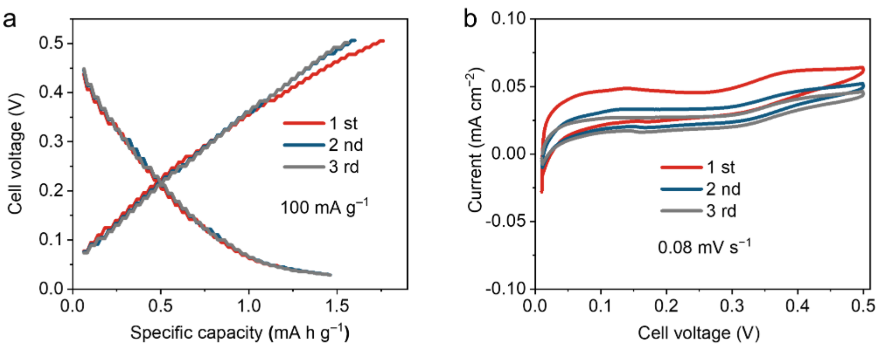

Supplementary Figure 11. The **a** GCD and **b** CV curves of pure porous carbon as the cathode. The pure porous carbon shows a negligible specific capacity of about 1.5 mAh g^{-1}.

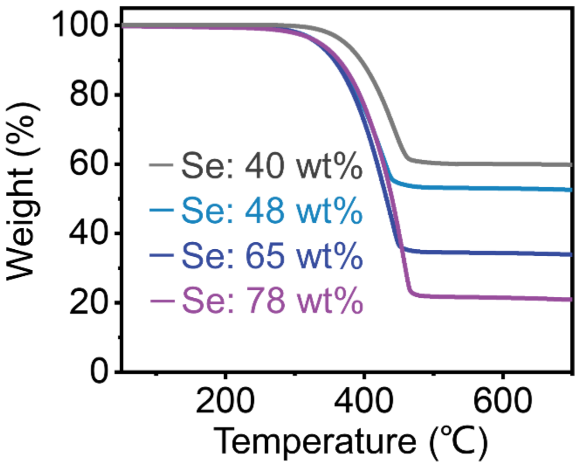

Supplementary Figure 12. TGA curves of Se@C composites with different Se contents.

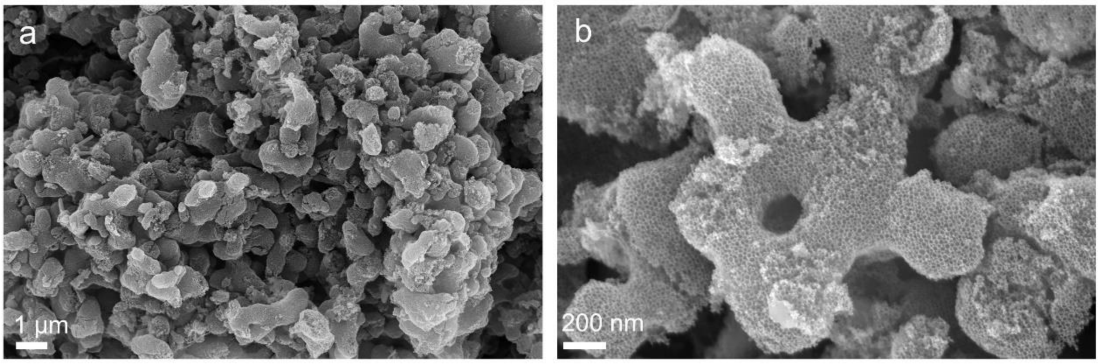

Supplementary Figure 13. SEM images of Se@C-40.

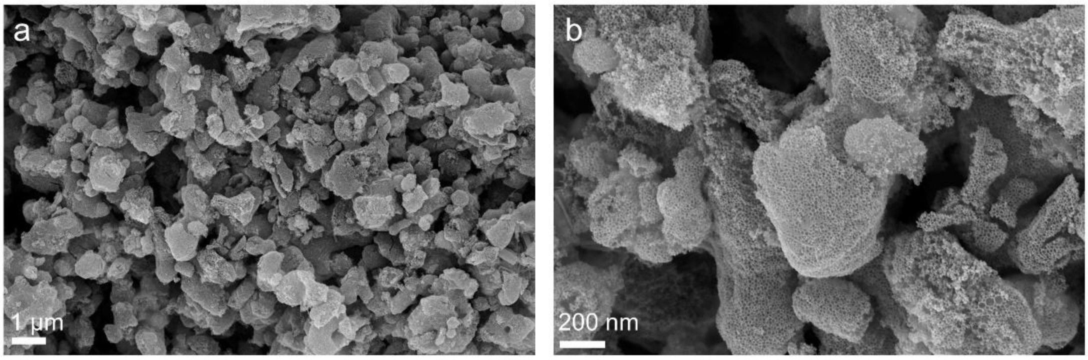

Supplementary Figure 14. SEM images of Se@C-65.

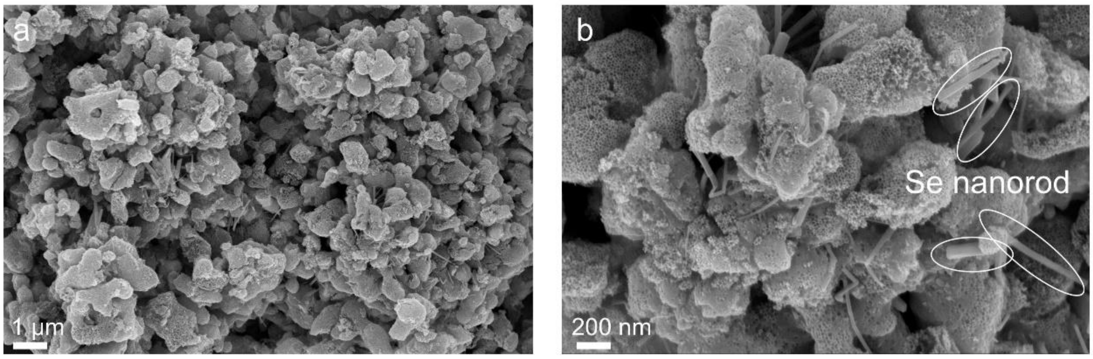

Supplementary Figure 15. SEM images of Se@C-78.

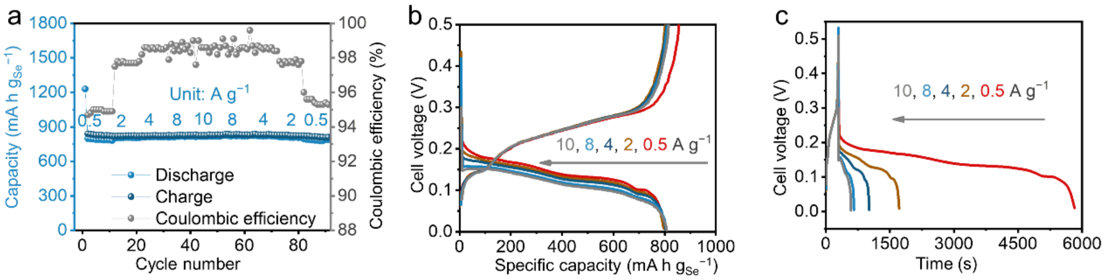

Supplementary Figure 16. The fast-charging rate performance of Cu|0.5 M $CuSO_4$|Se@C-40 cell: charging at 10 A g^{-1}, discharging at different currents.

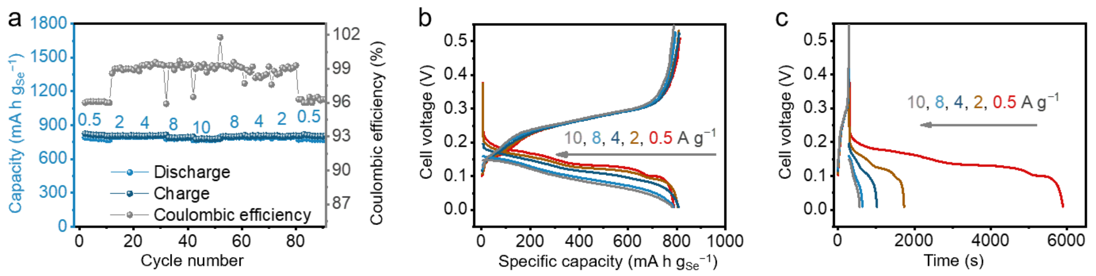

Supplementary Figure 17. The fast-charging rate performance of Cu|0.5 M $CuSO_4$|Se@C-65 cell: charging at 10 A g^{-1}, discharging at different currents.

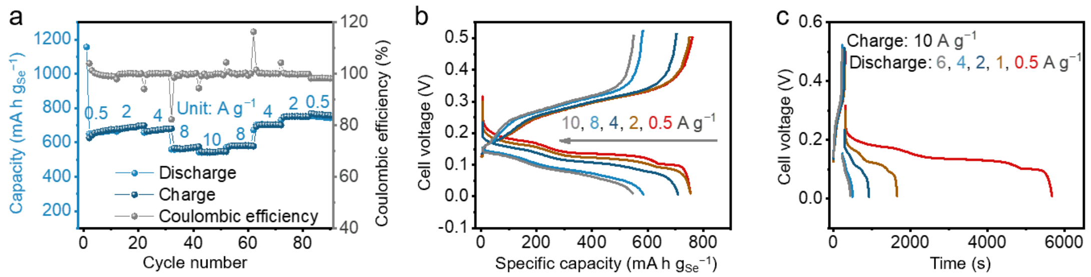

Supplementary Figure 18. The fast-charging rate performance of Cu|0.5 M $CuSO_4$|Se@C-78 cell: charging at 10 A g^{-1}, discharging at different currents.

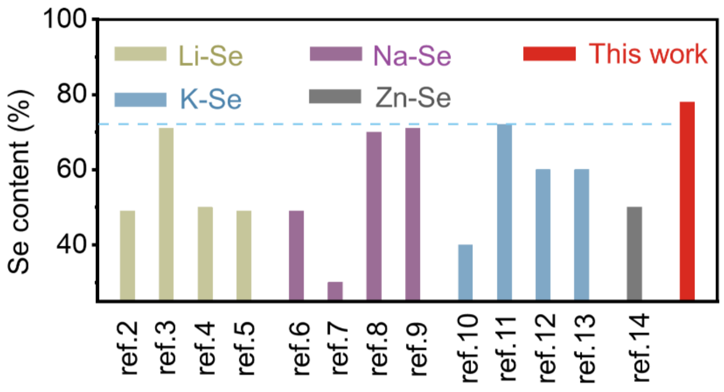

Supplementary Figure 19. The Se content comparison with other Se-based batteries reported in the literature.

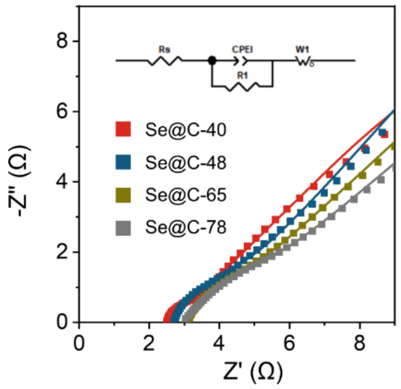

Supplementary Figure 20. The electrochemical impedance spectroscopy (EIS) measurements comparison of Cu|0.5 M $CuSO_4$|Se@C-40, Cu|0.5 M $CuSO_4$|Se@C-48, Cu|0.5 M $CuSO_4$|Se@C-65, and Cu|0.5 M $CuSO_4$|Se@C-78 cells. Inset shows the corresponding equivalent circuit. The points and lines represent measured and fitted curves, respectively. The fitted impendence parameters and errors are shown in Supplementary Table 2.

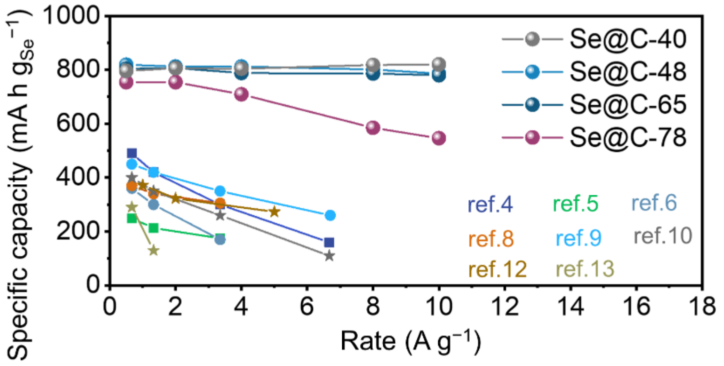

Supplementary Figure 21. The rate performance comparison with other Se-based batteries reported in the literature.

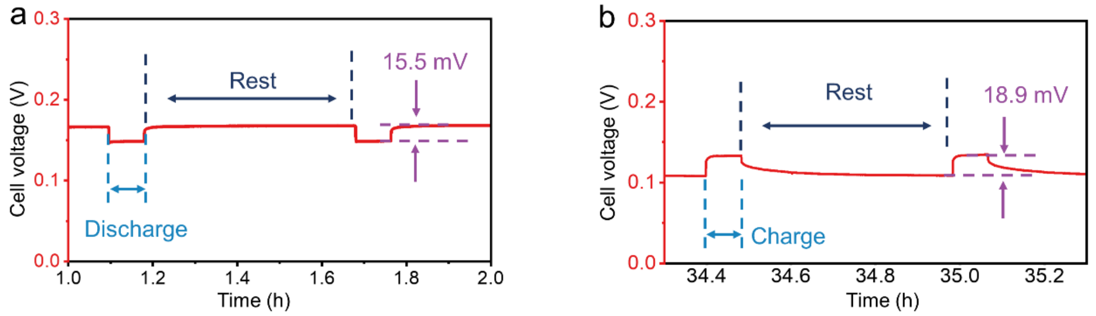

Supplementary Figure 22. Galvanostatic intermittent titration technique (GITT) curves during **a** discharging and **b** charging processes show low gaps between each polarization potentials (150 mV for discharging process and 126.9 mV for charging process) and each quasi-equilibrium potentials (165.5 mV for discharging process and 108 mV for charging process).

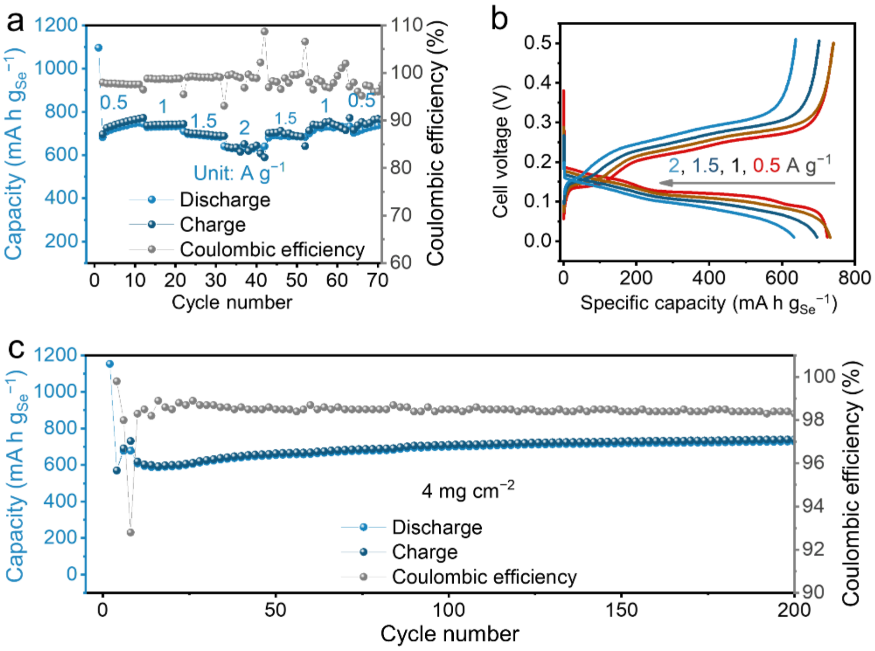

Supplementary Figure 23. The rate and cycling performance (1 A g^{-1}) and of Cu|0.5 M $CuSO_4$|Se@C-78 cell at areal loading of about 4 mg cm^{-2}.

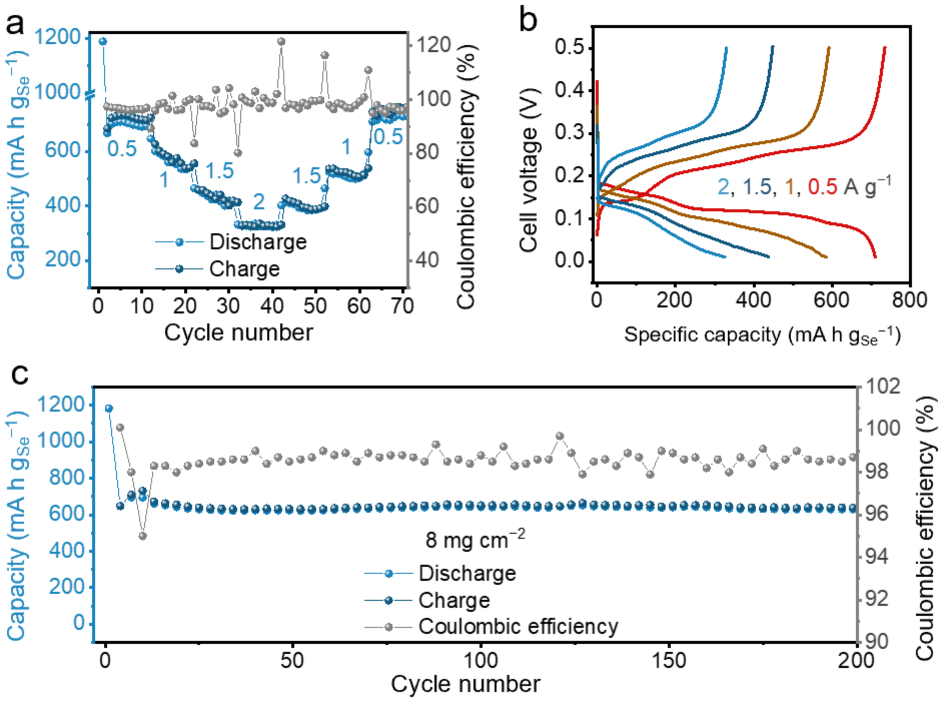

Supplementary Figure 24. The rate and cycling performance and of Cu|0.5 M $CuSO_4$|Se@C-78 cell at areal loading of about 8 mg cm^{-2}.

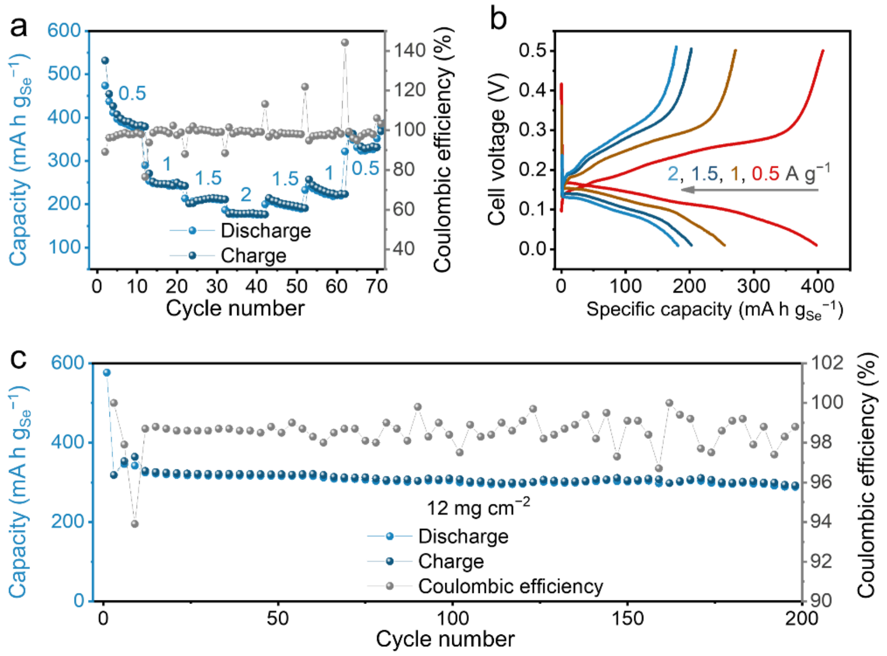

Supplementary Figure 25. The rate and cycling performance and of Cu|0.5 M $CuSO_4$|Se@C-78 cell at areal loading of about 12 mg cm^{-2}.

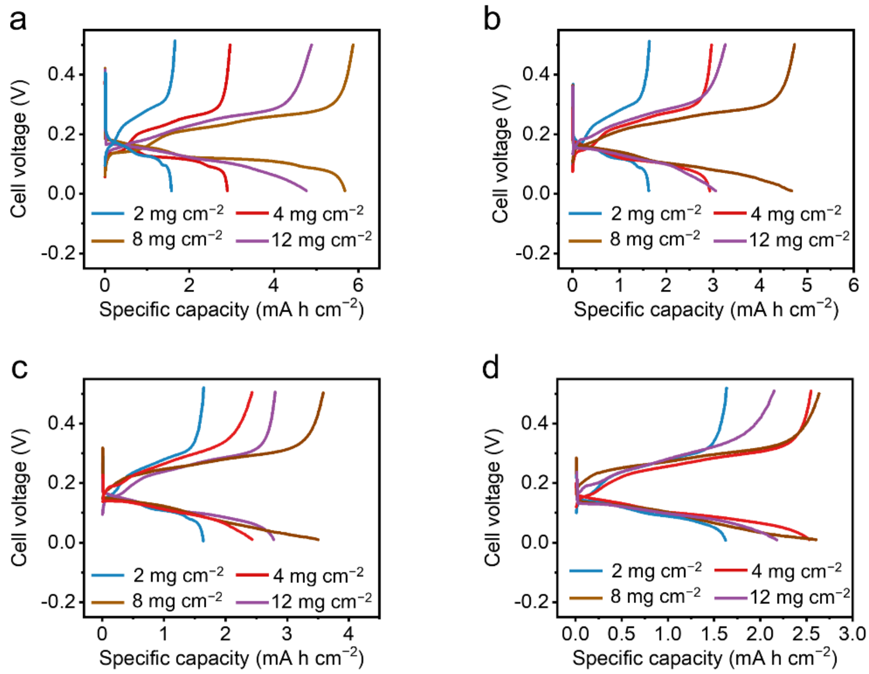

Supplementary Figure 26. The areal performances of Cu|0.5 M $CuSO_4$|Se@C-78 cell with different areal loadings at **a** 0.5, **b** 1, **c** 1.5, and **d** 2 A g^{-1}.

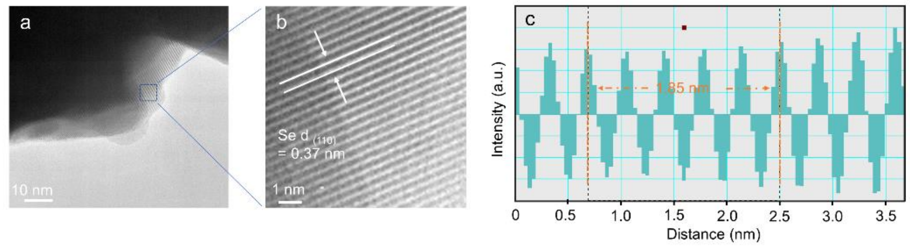

Supplementary Figure 27. TEM image of the final charging product in the in situ XRD measurements (in Cu|0.5 M $CuSO_4$|Se@C-48 coin cell). Se is observed in the final charging product, suggesting the reversibility of the four-electron Se chemistry.

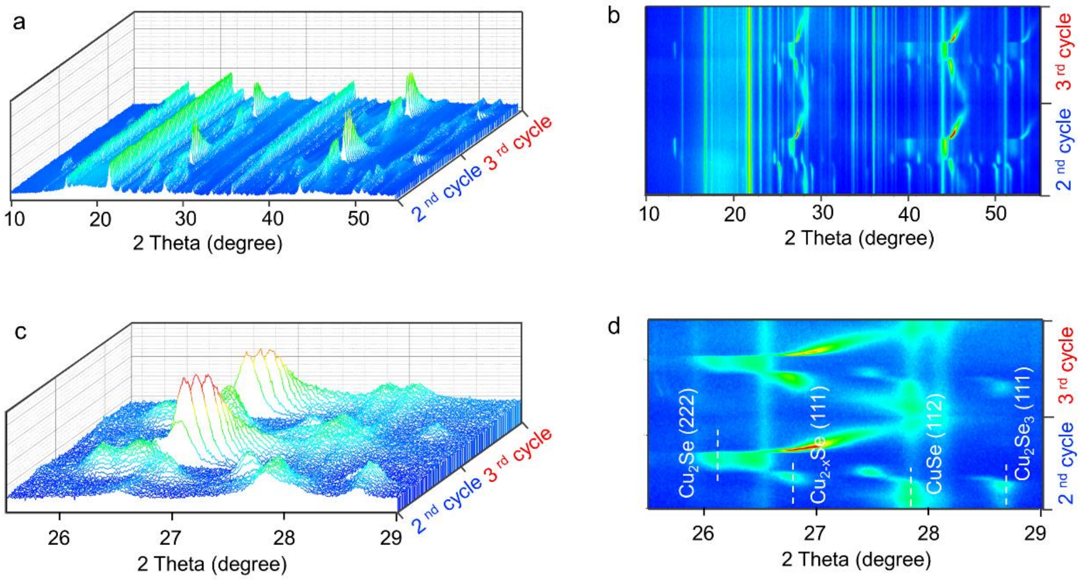

Supplementary Figure 28. In situ XRD of the second and third cycle discharging-charging process (in Cu|0.5 M $CuSO_4$|Se@C-48 coin cell). **a-b** show the full patterns between 10 and 55°. **c-d** show XRD patterns between 25.5 and 29° to clearly present the sequential conversion of Se to CuSe, Cu_3Se_2, $Cu_{2-x}Se$, and Cu_2Se during the discharging process.

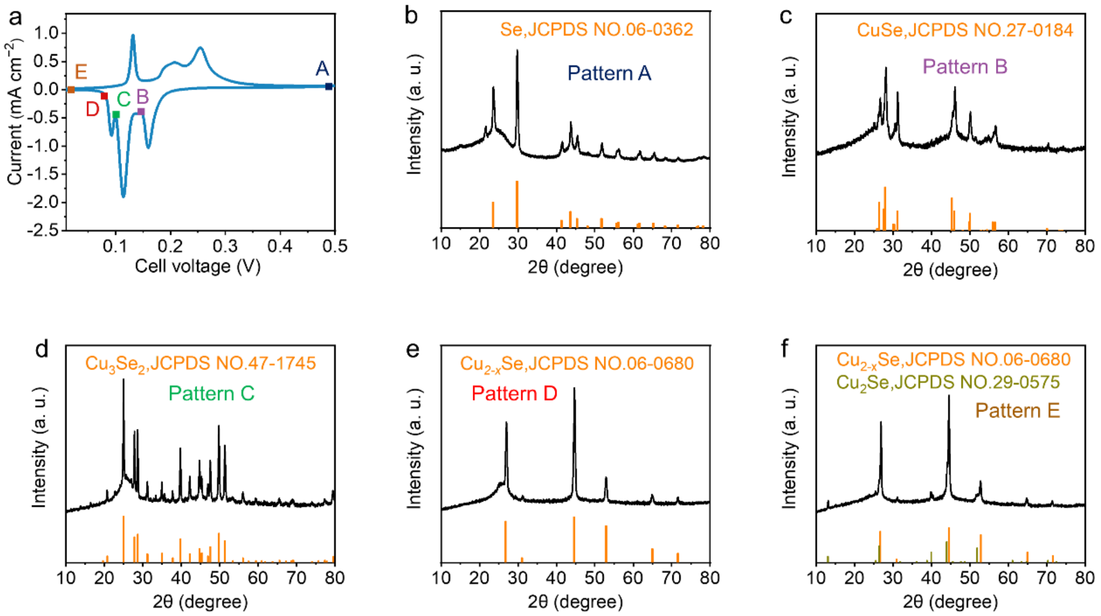

Supplementary Figure 29. Ex situ XRD patterns of discharging products. **a** The typical CV curve of Cu|0.5 M $CuSO_4$|Se@C-48 coin cell at a scan rate of 0.02 mV s^{-1}. XRD patterns of sample (**b**) before tested, at (**c**) the first reduction peak, (**d**) second reduction peak, (**e**) third reduction peak, and (**f**) 0.01 V *vs.* Cu^{2+}/Cu.

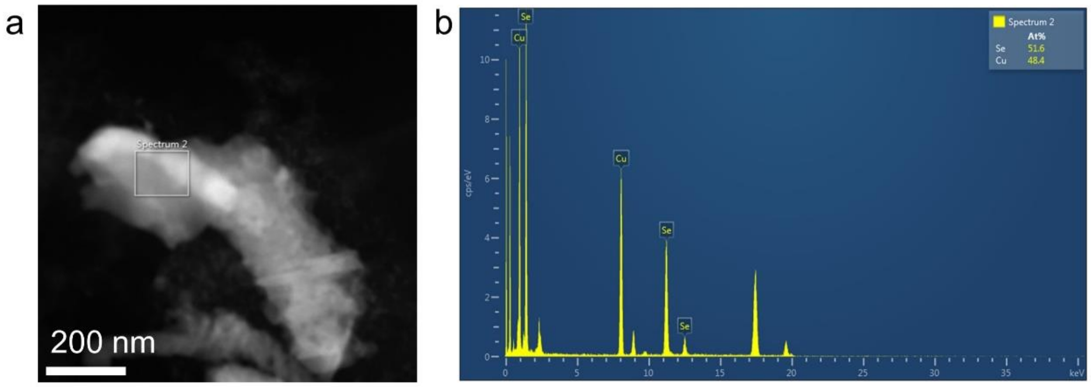

Supplementary Figure 30. TEM image and corresponding EDS spectrum of CuSe.

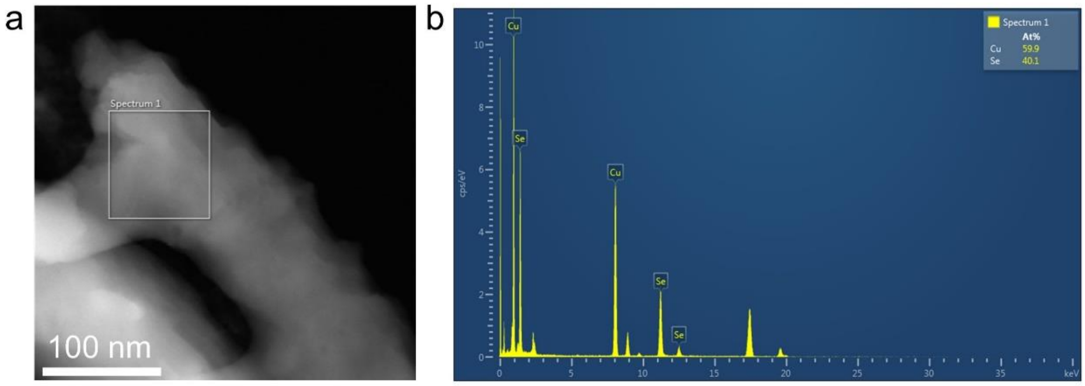

Supplementary Figure 31. TEM image and corresponding EDS spectrum of Cu_3Se_2.

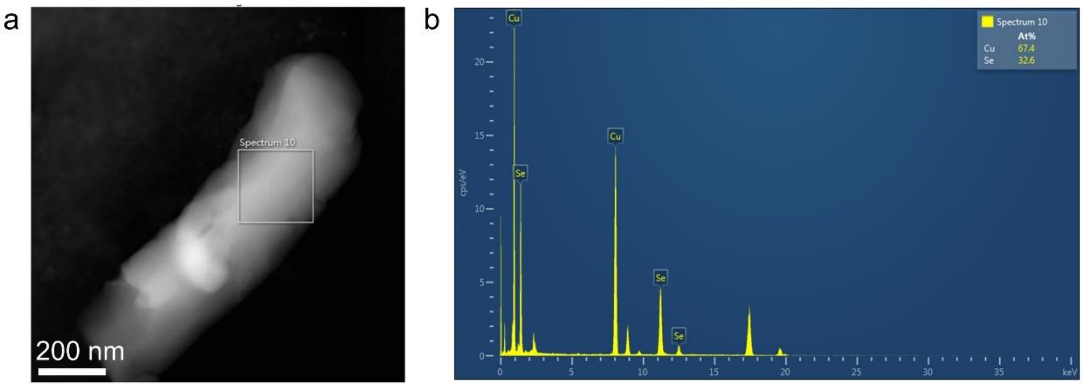

Supplementary Figure 32. TEM image and corresponding EDS spectrum of $Cu_{2\text{-}x}Se$.

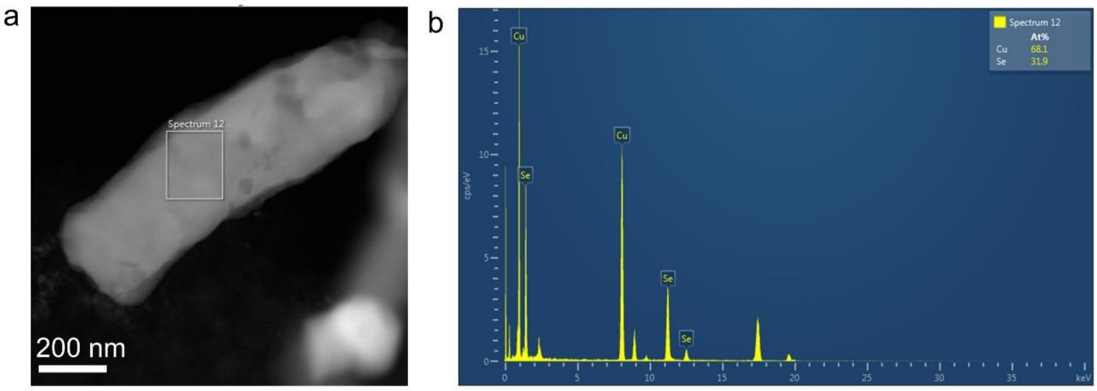

Supplementary Figure 33. TEM image and corresponding EDS spectrum of Cu_2Se.

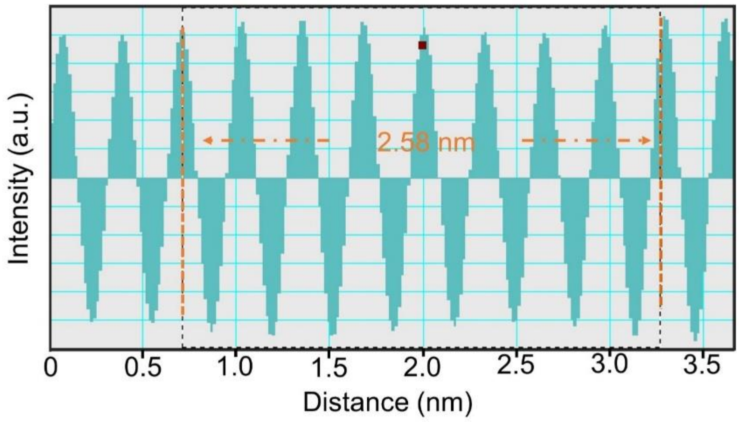

Supplementary Figure 34. Lattice spacing analysis of CuSe.

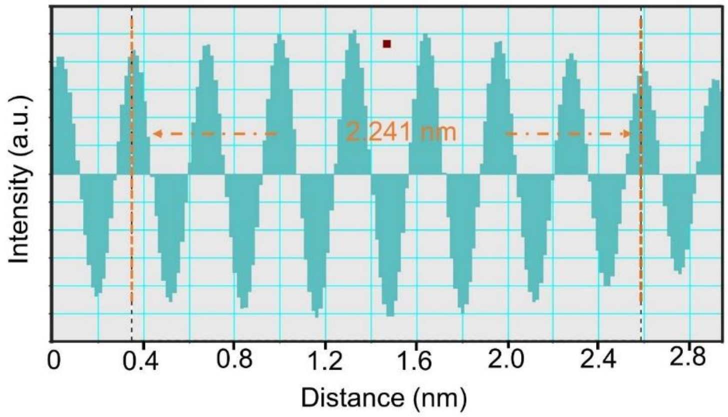

Supplementary Figure 35. Lattice spacing analysis of Cu_3Se_2.

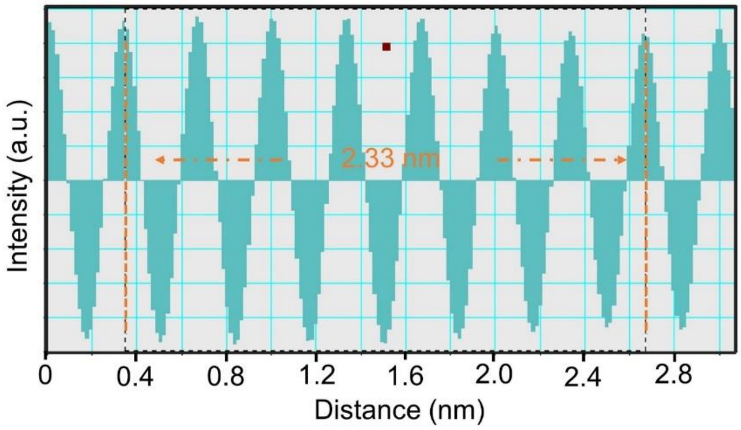

Supplementary Figure 36. Lattice spacing analysis of $Cu_{2-x}Se$.

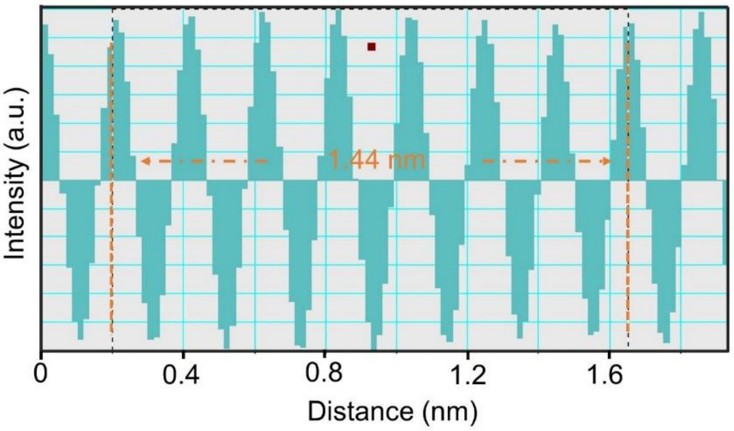

Supplementary Figure 37. Lattice spacing analysis of Cu_2Se.

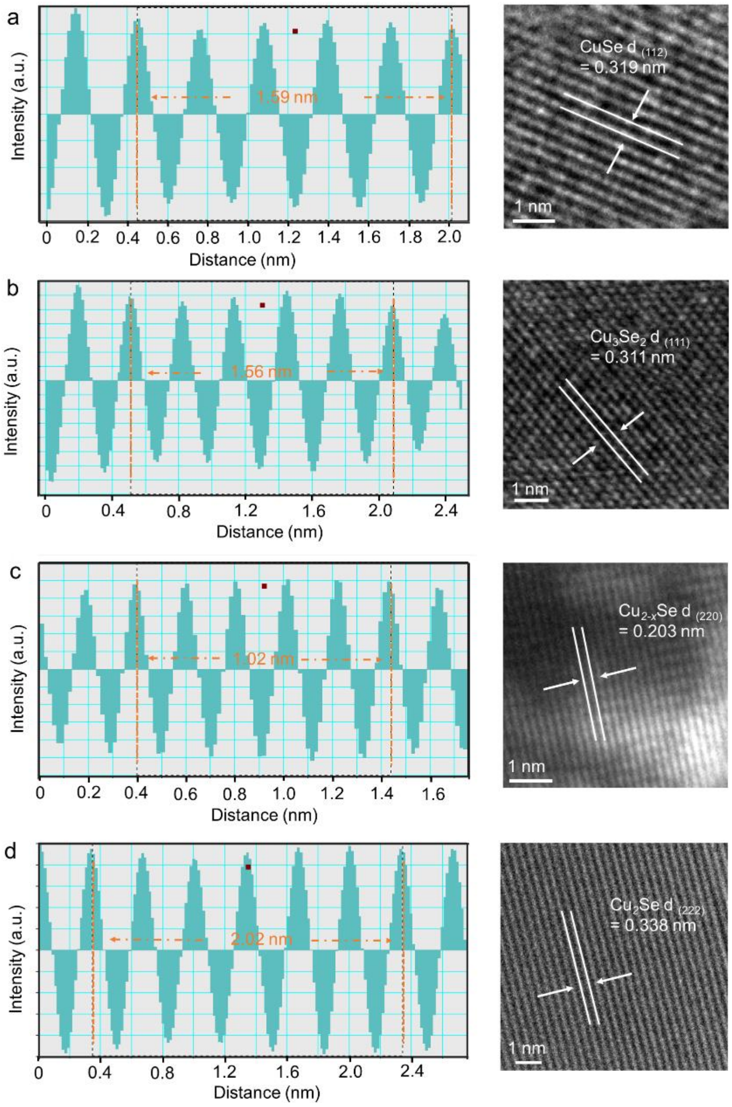

Supplementary Figure 38. High-resolution TEM images of four discharging products show their inter-planar spacings of 0.319, 0.311, 0.203, and 0.338 nm that can be assigned to the **a** (112) plane of CuSe, **b** (111) plane of Cu_3Se_2, **c** (220) plane of $Cu_{2-x}Se$, and **d** (222) plane of Cu_2Se, respectively.

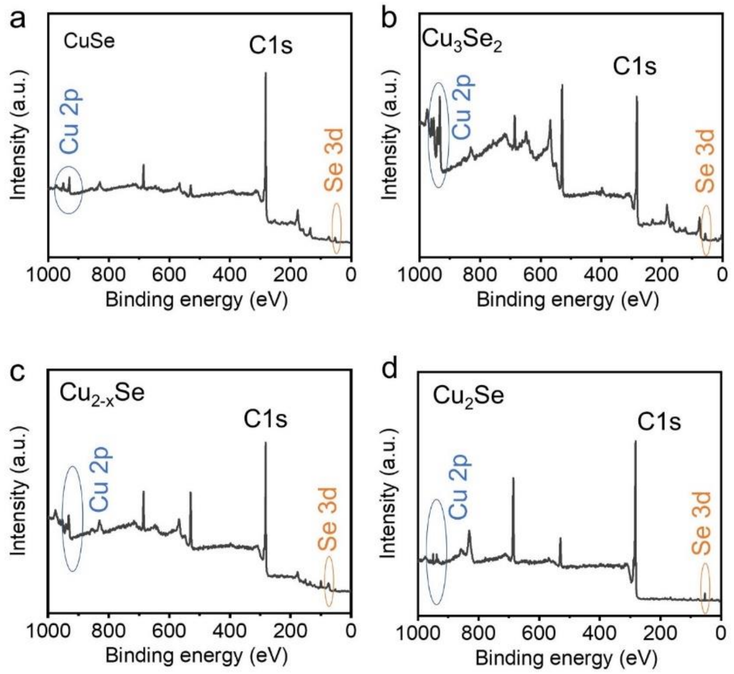

Supplementary Figure 39. XPS total survey spectrums of **a** CuSe, **b** Cu_3Se_2, **c** $Cu_{2-x}Se$, and **d** Cu_2Se.

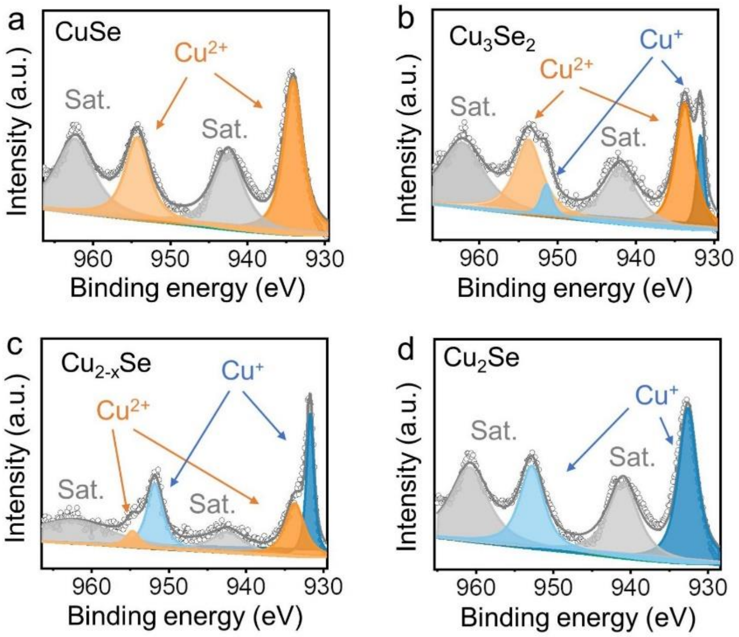

Supplementary Figure 40. High-resolution XPS spectra study of discharging products at different states. Cu peaks of **a** CuSe, **b** Cu_3Se_2, **c** $Cu_{2-x}Se$, and **d** Cu_2Se. The peaks at about 942.5 and 962.3 eV are their satellite peaks, denoted as “Sat.”.

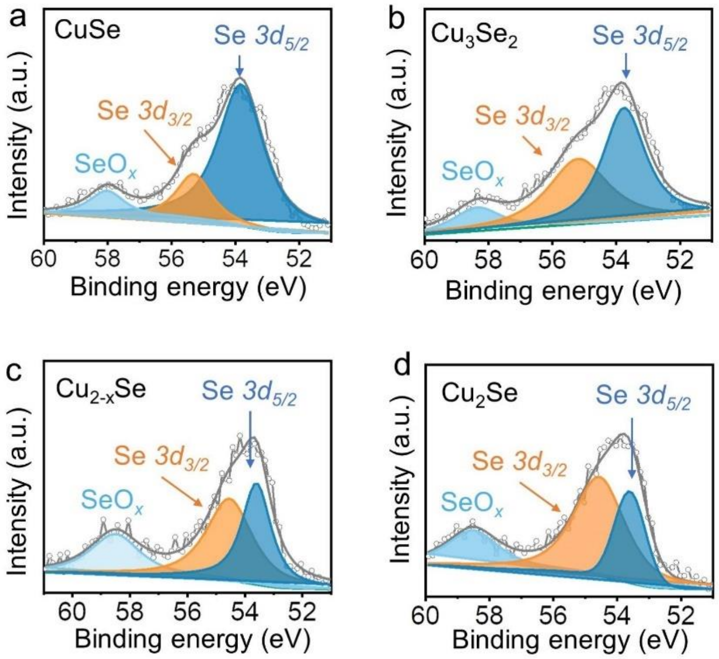

Supplementary Figure 41. High-resolution XPS spectra study of discharging products at different states. Se peaks of **a** CuSe, **b** Cu_3Se_2, **c** $Cu_{2-x}Se$, and **d** Cu_2Se. All Se spectrum show three main peaks, corresponding to Se $3d_{5/2}$,$3d_{3/2}$, and SeO_x, respectively.

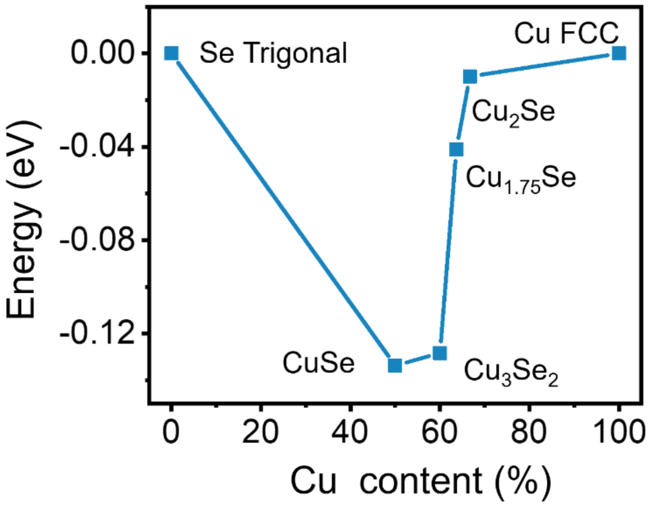

Supplementary Figure 42. Formation energy versus copper concentration.

Formation Energy is defined as

$$E_f = E_i - xE_{Cu} - (1-x)E_{Se},$$

where x is copper concentration, and E_i, E_{Cu}, and E_{Se} represent total energy per atom of System i (i= Se, CuSe, Cu_3Se_2, $Cu_{2-x}Se$, Cu_2Se, and Cu), total energy per atom of

face centered cubic (FCC) Cu, and that of trigonal Se, respectively. The formation energies for all systems are negative, suggesting these phases could exist at given copper concentration.

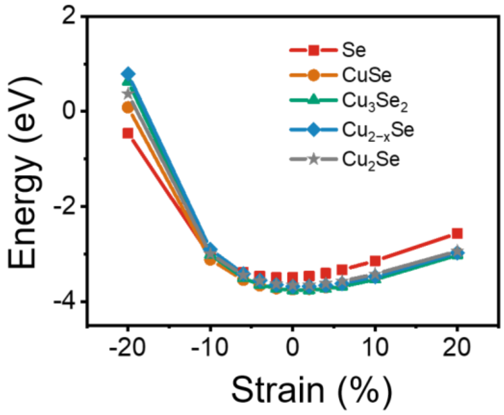

Supplementary Figure 43. The corresponding total energy with respect to strain.

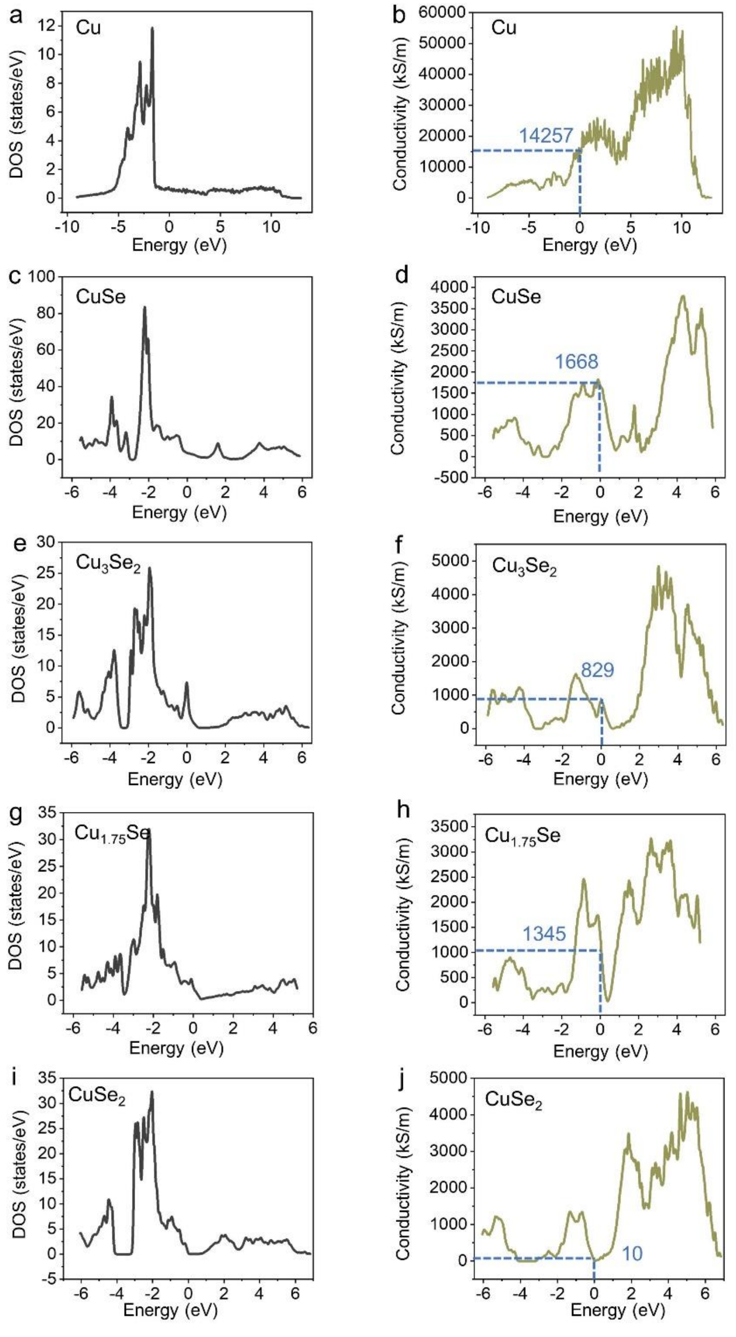

Supplementary Figure 44. Density of states (DOS) per unit cell and conductivity for **a-b** Cu, **c-d** CuSe, **e-f** Cu_3Se_2, **g-h** $Cu_{1.75}Se$, and **i-j** Cu_2Se. Vienna Ab-initio Simulation Package (VASP) is used to obtain band structures followed by conductivity calculation implemented in BoltzTrap2[1] based on Boltzmann transport equation. Relaxation time takes 10^{-14} s in all conductivity calculations.

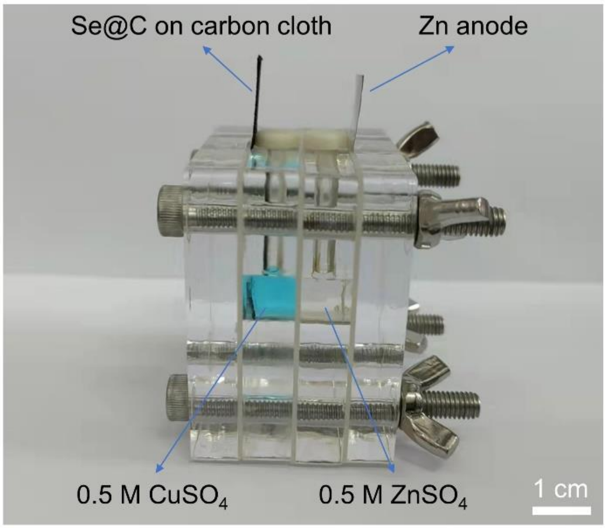

Supplementary Figure 45. The photographic picture of the home-made Zn||Se@C-48 full cell.

Supplementary Table 1. The comparisons with other Se-based batteries.

Batteries	Potential	Electrolyte	Se content	Max Capacity	Rate performance	Areal Capacity	Plateau slope (V/ (1000 mAh g^{-1}))	cell charge/discharge voltage hysteresis	Decay rate	Reference
Zn-Se	~ 0.5 V vs. SHE	Aqueous $CuSO_4$	48%; 65%; 78%	1298 mAh g^{-1} at 500 mA g^{-1}	758 mAh g^{-1} at 10 A g^{-1}	5.6 mAh cm^{-2} (8 mg cm^{-2})	0.11 at 0.5 A g^{-1}	0.11 at 0.5 A g^{-1}	0.017% at 5 A g^{-1}	**This work**
Li-Se	~ – 1.2 V vs. SHE	1 M $LiPF_6$ in EC/DMC	49%	670 mAh g^{-1} at 67 mA g^{-1}	~ 270 mAh g^{-1} at 3.3 A g^{-1}	1.34 mAh cm^{-2} (2 mg cm^{-2})	0.6 at 0.067 A g^{-1}	~0.3 V at 0.067 A g^{-1}	0.70 % at 0.67 A g^{-1}	2
Li-Se	~ – 1.1 V vs. SHE	1 wt% $LiNO_3$ in 1,3-dioxolane /DME	73%	613 mAh g^{-1} at 67 mA g^{-1}	~ 420 mAh g^{-1} at 6.7 A g^{-1} ~ 311 mAh g^{-1} at 33 A g^{-1}	0.49 mAh cm^{-2} (0.8 mg cm^{-2})	1.11 at 0.067 A g^{-1}	~0.25 V at 0.067 A g^{-1}	0.145 % at 0.33 A g^{-1}	3
Li-Se	~ – 1.2 V vs. SHE	1 M $LiPF_6$ in EC/DMC	50%	585 mAh g^{-1} at 135 mA g^{-1}	~ 155 mAh g^{-1} at 6.7 A g^{-1}	0.88 mAh cm^{-2} (1.5 mg cm^{-2})	1.18 at 0.067 A g^{-1}	~0.25 V at 0.13 A g^{-1}	0.02 % at 0.13 A g^{-1}	4
Li-Se	~ – 1.2 V vs. SHE	1 M $LiPF_6$ in EC/DMC	49%	588 mAh g^{-1} at 338 mA g^{-1}	~ 175 mAh g^{-1} at 3.3 A g^{-1}	N/A	1.05 at 0.33 A g^{-1}	~0.4 V at 0.33 A g^{-1}	0.11 % at 0.33 A g^{-1}	5
Na-Se	~ – 1.5 V vs. SHE	1 M $NaClO_4$ in EC/DMC	30%	485 mAh g^{-1} at 67 mA g^{-1}	~ 150 mAh g^{-1} at 3.3 A g^{-1}	N/A	1.22 at 0.167 A g^{-}	~0.3 V at 0.167 A g^{-1}	0.078 % at 0.17 A g^{-1}	6
Na-Se	~ – 1.2 V vs. SHE	1 M $NaPF_6$ in EC/EMC	~70 %	620 mAh g^{-1} at 50 mA g^{-1}	~620 mAh g^{-1} at 0.05 A g^{-1}	N/A	0.8 at 0.01 A g^{-1}	~0.55 V at 0.01 A g^{-1}	0.72 % at 0.01 A g^{-1}	7
Na-Se	~ – 1.1 V vs. SHE	1 M $NaClO_4$ in PC+5% FEC	71%	577 mAh g^{-1} at 100 mA g^{-1}	~320 mAh g^{-1} at 5 A g^{-1}	N/A	3 at 0.1 A g^{-1}	~0.4 V at 0.1 A g^{-1}	0.012 % at 2 A g^{-1}	8
Na-Se	~ – 1.2 V vs. SHE	1 M $NaClO_4$ in EC/DMC	53%	583 mAh g^{-1} at 67 mA g^{-1}	~184 mAh g^{-1} at 13.6 A g^{-1}	1.16 mAh cm^{-2} (2 mg cm^{-2})	1.0 at 0.13 A g^{-1}	~0.3 V at 0.13 A g^{-1}	0.023 % at 0.13 A g^{-1}	9
K-Se	~ – 1.7 V vs. SHE	1 M KPF_6 in EC/PC	40%	652 mAh g^{-1} at 67 mA g^{-1}	~140 mAh g^{-1} at 6.7 A g^{-1}	0.33 mAh cm^{-2} (0.5 mg cm^{-2})	1.67 at 0.13 A g^{-1}	~0.45 V at 0.067 A g^{-1}	N/A	10
K-Se	~ – 1.1 V vs. SHE	KTFSI in DEGDME	72%	684 mAh g^{-1} at 13.5 mA g^{-1}	~150 mAh g^{-1} at 0.67 A g^{-1}	1.12 mAh cm^{-2} (2.8 mg cm^{-2})	1.87 at 0.13 A g^{-1}	~0.45 V at 0.067 A g^{-1}	0.293 % at 0.067 A g^{-1}	11
K-Se	~ – 1.6 V vs. SHE	0.7 M KPF_6 in EC/DMC	60%	585 mAh g^{-1} at 100 mA g^{-1}	~273 mAh g^{-1} at 5 A g^{-1}	0.877 mAh cm^{-2} (1.5 mg cm^{-2})	1.87 at 0.1 A g^{-1}	~0.55 V at 0.1 A g^{-1}	0.029 % at 0.8A g^{-1}	12
K-Se	~ – 1.5 V vs. SHE	0.7 M KPF_6 in EC/DMC	~ 60%	492 mAh g^{-1} at 135 mA g^{-1}	~137 mAh g^{-1} at 1.36 A g^{-1}	1.03 mAh cm^{-2} (2.1 mg cm^{-2})	1.07 at 0.13 A g^{-1}	~0.7 V at 0.13 A g^{-1}	0.06 % at 0.13A g^{-1}	13
Zn-Se	~ 0.27 vs. SHE	1 M ZnTFSI in water	~ 50%	611 mAh g^{-1} at 100 mA g^{-1}	~315 mAh g^{-1} at 5 A g^{-1}	1.2 mAh cm^{-2} (2.1 mg cm^{-2})	0.61 at 0.1 A g^{-}	~0.4 V at 0.1 A g^{-1}	0.02 % at 1A g^{-1}	14

Supplementary Table 2. Fitted impendence parameters of Cu|0.5 M $CuSO_4$|Se@C coin cells. Rs , Rct , CPE, W1 represent series resistance, charge-transfer resistance, constant phase element, and Warburg impedance respectively.

Cells	Equivalent circuit	Rs	Error of fitted Rs	R1	Error of fitted R1
Cu|0.5 M $CuSO_4$|Se@C-40 coin cell	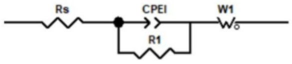	1.21	1.2%	0.21	7.3%
Cu|0.5 M $CuSO_4$|Se@C-48 coin cell	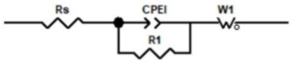	2.61	0.72%	0.61	5.4%
Cu|0.5 M $CuSO_4$|Se@C-65 coin cell	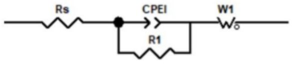	2.92	0.74%	0.56	7.4%
Cu|0.5 M $CuSO_4$|Se@C-78 coin cell	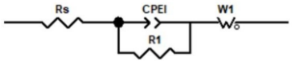	2.90	0.76%	0.95	7.0%

References

1. Madsen G.K.H., Carrete J., Verstraete M.J. BoltzTraP2, a program for interpolating band structures and calculating semi-classical transport coefficients. *Comput. Phys. Commun.* **231**, 140-145 (2018).
2. Yang C.P., Xin S., Yin Y.X., Ye H., Zhang J., Guo Y.G. An Advanced Selenium-Carbon Cathode for Rechargeable Lithium-Selenium Batteries. *Angew. Chem. Int. Ed.* **52**, 8363-8367 (2013).
3. Tian H., Tian H., Wang S., Chen S., Wang G. High-power lithium-selenium batteries enabled by atomic cobalt electrocatalyst in hollow carbon cathode. *Nat. Commun.* **11**, 1-12 (2020).
4. Kalimuthu B., Nallathamby K. Optimization of Structure and Porosity of Nitrogen Containing Mesoporous Carbon Spheres for Effective Selenium Confinement in Futuristic Lithium-Selenium Batteries. *ACS Sustain. Chem. Eng.* **6**, 7064-7077 (2018).
5. Liu T., *et al.* Selenium Embedded in Metal-Organic Framework Derived Hollow Hierarchical Porous Carbon Spheres for Advanced Lithium-Selenium Batteries. *ACS Appl. Mater. Interfaces* **8**, 16063-16070 (2016).
6. Luo C., Xu Y., Zhu Y., Liu Y., Wang C. Selenium@mesoporous carbon composite with superior lithium and sodium storage capacity. *ACS Nano* **7**, 8003-8010 (2013).
7. Ali, *et al.* A New Class of Lithium and Sodium Rechargeable Batteries Based on Selenium and Selenium-Sulfur as a Positive Electrode. *J. Am. Chem. Soc.* **134**, 4505–4508 (2012).
8. Yang X., *et al.* Vapor-Infiltration Approach toward Selenium/Reduced Graphene Oxide Composites Enabling Stable and High-Capacity Sodium Storage. *ACS Nano* **12**, 7397-7405 (2018).
9. Ding J., *et al.* Exceptional energy and new insight with a sodium-selenium battery based on a carbon nanosheet cathode and a pseudographite anode. *Energy Environ. Sci.* **10**, 153-165 (2017).
10. Liu Y., *et al.* A new energy storage system: Rechargeable potassium-selenium battery. *Nano Energy* **35**, 36-43 (2017).
11. Liu Q., Deng W., Pan Y., Sun C.F. Approaching the voltage and energy density limits of potassium-selenium battery chemistry in a concentrated ether-based electrolyte. *Chem Sci.* **11**, 6045-6052 (2020).
12. Yu Y., *et al.* CNT Interwoven Nitrogen and Oxygen Dual-Doped Porous Carbon Nanosheets as Free-Standing Electrodes for High-Performance Na-Se and K-Se Flexible Batteries. *Adv. Mater.* **30**, 1805234 (2018).
13. Kim J.K., Kang Y.C. Encapsulation of Se into Hierarchically Porous Carbon Microspheres with Optimized Pore Structure for Advanced Na-Se and K-Se Batteries. *ACS Nano* **14**, 13203-13216 (2020).
14. Chen Z., *et al.* Zinc/selenium conversion battery: a system highly compatible with both organic and aqueous electrolytes. *Energy Environ. Sci.* **14**, 2441-2450 (2021).
